# Early Flexible Sigmoidoscopy Improves Clinical Outcomes in Acute Severe Ulcerative Colitis

**DOI:** 10.1093/crocol/otad032

**Published:** 2023-05-31

**Authors:** Shreyak Sharma, Darrick K Li, Louis J Levine, Abdelkader Chaar, Chandler McMillan, Jill K J Gaidos, Deborah D Proctor, Badr Al-Bawardy

**Affiliations:** Department of Internal Medicine, Yale School of Medicine, New Haven, Connecticut, USA; Section of Digestive Diseases, Yale School of Medicine, New Haven, Connecticut, USA; Department of Internal Medicine, Yale School of Medicine, New Haven, Connecticut, USA; Department of Internal Medicine, Yale School of Medicine, New Haven, Connecticut, USA; Yale School of Medicine, New Haven, Connecticut, USA; Section of Digestive Diseases, Yale School of Medicine, New Haven, Connecticut, USA; Section of Digestive Diseases, Yale School of Medicine, New Haven, Connecticut, USA; Section of Digestive Diseases, Yale School of Medicine, New Haven, Connecticut, USA; Department of Internal Medicine, Division of Gastroenterology and Hepatology, King Faisal Specialist Hospital and Research Center, Riyadh, Saudi Arabia

**Keywords:** ulcerative colitis, IBD, flexible sigmoidoscopy, infliximab

## Abstract

**Objectives:**

Guidelines recommend performing a flexible sigmoidoscopy in patients hospitalized with acute severe ulcerative colitis (ASUC). However, it is unclear if time to sigmoidoscopy affects relevant clinical outcomes. We aimed to assess the impact of early sigmoidoscopy on clinical outcomes using a well-characterized cohort of patients with ASUC.

**Methods:**

This is a single-center, retrospective study of all patients hospitalized with ASUC from January 1, 2012 to November 1, 2021. Early sigmoidoscopy was defined as occurring within 72 hours of admission while delayed sigmoidoscopy was defined as occurring >72 hours after admission. Primary outcomes were cumulative days of intravenous (IV) corticosteroid (CS) use, length of hospital stay, and colectomy rates. Secondary outcomes were time to infliximab (IFX) rescue and inpatient opioid medication use.

**Results:**

A total of 112 patients hospitalized with ASUC who underwent sigmoidoscopy were included in the analysis. Eighty-seven patients (78%) had early sigmoidoscopy and 25 (22%) had delayed sigmoidoscopy. Patients in the early sigmoidoscopy group were exposed to significantly fewer days of IV CS (4.5 vs 9.2 days; *P* < .001), had shorter hospital stays (6.4 vs 19.3 days; *P* < .001), and shorter time to IFX rescue (3.5 vs 6.4 days; *P* = .004). Rates of colectomy in the early and delayed sigmoidoscopy groups were 17% versus 28%, respectively (*P* = .23). Longer time to sigmoidoscopy was associated with a 16% increased risk of colectomy (HR = 1.16, *P* = .002).

**Conclusions:**

In this well-characterized cohort, early sigmoidoscopy in ASUC was associated with favorable clinical outcomes. These findings highlight the benefits of early sigmoidoscopy in patients with ASUC. Larger prospective studies are needed to corroborate these findings.

## Introduction

Ulcerative colitis (UC) is a chronic inflammatory bowel disease (IBD) characterized by relapsing symptoms of bloody diarrhea and abdominal pain. The global burden of UC is increasing and is associated with increased healthcare costs.^1^ IBD is estimated to account for over $6 billion of annual direct healthcare expenditure in the United States and over €5 billion in Europe.^2–4^ Hospitalizations are one of the major drivers of increased healthcare cost in UC.^5^

Acute severe ulcerative colitis (ASUC) is defined by the Truelove and Witts criteria as the following: bloody stool frequency > 6/day; AND one of the following as evidence of systemic toxicity: temperature > 37.8°C, pulse > 90 beats/min, hemoglobin < 10.5 g/dL, erythrocyte sedimentation rate (ESR) > 30 mm/h.^6^ The rates of hospitalization due to ASUC remain significant despite multiple advances in treatment modalities and evolution of treatment targets. Various studies suggest a 20%–30% prevalence of an acute severe flare in patients with UC.^7,8^ More recently, a systematic review and meta-analysis showed that the 5-year risk of UC hospitalization can be as high as 21.5%.^9^ Initial management of ASUC involves performing a flexible sigmoidoscopy to grade the severity of inflammation and to obtain biopsies to rule out cytomegalovirus virus (CMV) infection. Endoscopic evaluation has important prognostic value in this setting as the presence of large/deep ulcers is associated with a high likelihood of treatment failure with intravenous (IV) corticosteroids and need for colectomy.^10^

Consensus guidelines from the American College of Gastroenterology (ACG), European, British, and Canadian gastroenterology societies recommend a flexible sigmoidoscopy after admission to assess disease severity, rule out CMV and other causes of symptoms including ischemic colitis.^11–14^ However, not all guidelines offer a recommendation regarding the specific timing of the flexible sigmoidoscopy in ASUC. This is primarily driven by a lack of data on outcomes with an early versus delayed flexible sigmoidoscopy. Two studies using data from the National Inpatient Sample (NIS) have found that early endoscopy is associated with a reduction in hospital length of stay, hospital cost, and mortality.^15,16^ However, these studies were based on coding data. Moreover, the severity of UC flares, baseline disease activity, and medical therapy were not elucidated in these studies. The purpose of this study was to evaluate whether early flexible sigmoidoscopy is associated with favorable outcomes in patients with ASUC compared to delayed flexible sigmoidoscopy in a well-characterized cohort.

## Methods

### Study Population, Variable Definitions, Outcomes

This study is a single-center, retrospective review of all UC patients who were admitted with ASUC from January 1, 2012 to November 1, 2021. The study protocol was approved by the Yale University Institutional Review Board (IRB# 2000031140). We included all adult (age ≥ 18 years) patients who were admitted to the hospital for ASUC and underwent a flexible sigmoidoscopy during the hospital admission. Determination of ASUC was based on the modified Truelove and Witts’ clinical criteria: bloody stool frequency > 6/day and one of the following as evidence of systemic toxicity: temperature > 37.8°C, pulse > 90 beats/min, hemoglobin < 10.5 g/dL, ESR > 30 mm/h. Exclusion criteria included lack of endoscopic evaluation, lack of confirmed UC diagnosis, and patients with UC hospitalized for indications other than ASUC.

Demographic information at time of admission including age, sex, body mass index, and smoking status were extracted from each individual’s medical record to form the data set. Disease-related variables including date of UC diagnosis, disease location, and presence of documented extraintestinal manifestations were noted. Other collected variables included all current and prior medical therapies for UC, dates and times of hospital admission, flexible sigmoidoscopy findings and timing, presence of CMV by histopathology, or *Clostridioides difficile* (*C. difficile*) infection, labs including admission C-reactive protein (CRP) and albumin, and need for rescue medical therapy.

Early flexible sigmoidoscopy was defined as within 72 hours of admission while delayed flexible sigmoidoscopy was defined as >72 hours after admission based on the latest ACG guidelines.^14^ Variables were compared between the early and delayed flexible sigmoidoscopy groups. The primary outcomes were duration of IV corticosteroid use, length of hospital stay, and time to rescue medical therapy. Secondary outcomes included rates of colectomy and inpatient opioid medication use. The results of analyses using cutoffs for flexible sigmoidoscopy within 24 and 48 hours of admission were also performed are provided in Supplementary Material.

### Statistical Analysis

Continuous variables were analyzed using an unpaired Student’s *t*-test. Categorical variables were analyzed using a Pearson’s chi-square test. To develop multivariate regression models to assess the impact of time to flexible sigmoidoscopy on clinically meaningful outcomes, an exploratory univariate analysis was first performed. To develop our model, clinically plausible variables, and variables with *P* < .10 on univariate analysis were identified and incorporated as independent variables in multivariate regression models. However, no variables were significant on univariate analysis and as such clinically plausible variables based on clinical experience and the literature were used to build the model. The variables included in the model were time to flexible sigmoidoscopy, baseline CRP, Mayo UC endoscopic subscore of 3, anti-tumor necrosis factor (TNF) exposure, and use of oral corticosteroids on admission. Linear regression models were used to determine factors associated with duration of IV steroids and time to salvage infliximab. Cox proportional hazards multivariate regression modeling and Kaplan–Meier plots were used to model time to colectomy.

A *P*-value < .05 was considered statistically significant. JMP (SAS Institute Inc., Cary, NC) and SPSS Statistics for Windows, version 28.0 (IBM Corp., Armonk, NY) were used for data analysis.

## Results

### Demographics

A total of 112 patients with ASUC who underwent flexible sigmoidoscopy during the admission were included in the analysis. A total of 87 patients (78%) underwent early flexible sigmoidoscopy and 25 patients (22%) underwent delayed flexible sigmoidoscopy. The median time to flexible sigmoidoscopy in the entire cohort was 39.7 (interquartile range [IQR] 19.5–69.2) hours. The median time to flexible sigmoidoscopy in the early group was 34.3 (IQR 15.5–56.6) hours compared to 109.9 (IQR 92–144) hours in the delayed group. Reasons for delay in flexible sigmoidoscopy were: early weekend admission (36%, *n* = 9), outpatient flexible sigmoidoscopy performed in the past 30 days (24%, *n* = 6), treating physicians’ discretion (40%, n = 10). The mean age at admission was 39.2 ± 16.9 years and 54% of the patients were male. The median disease duration was 2 years with a range of 1–10 years. The early and delayed flexible sigmoidoscopy groups were similar in terms of baseline characteristics and disease activity (Table 1).

**Table 1.** Baseline characteristics

Variables	Early flexible sigmoidoscopy ≤ 72 hours (*n* = 87)	Delayed flexible sigmoidoscopy > 72 hours (*n* = 25)	*P*-value
Patient characteristics			
Age (years), mean (SD)	39.1 (16.5)	39.8 (18.4)	.84
Female, *n* (%)	39 (44.8)	12 (48)	.78
BMI, mean (SD)	26.9 (6.7)	25.3 (6.5)	.27
Current smoker, *n* (%)	3 (3.5)	3 (12)	.08
On IMM on admission, *n* (%)	3 (3.5)	3 (12)	.09
Biologic-naïve, *n* (%)	55 (63.2)	16 (64)	.94
Prior anti-TNF, *n* (%)	26 (29.9)	8 (32)	.84
On chronic opioids, *n* (%)	7 (8.1)	1 (4)	.49
On oral corticosteroids at time of admission, *n* (%)	44 (50.6)	12 (48)	.82
Disease activity			
Pancolitis, *n* (%)	60 (68.9)	20 (80)	.28
Presence of extraintestinal manifestations (EIM), *n* (%)	8 (9.2)	1 (4)	.39
Concomitant CMV infection, *n* (%)	2 (2.3)	1 (4)	.17
Concomitant *C. difficile* infection, *n* (%)	3 (3.5)	1 (4)	.89
C-reactive protein md/dL at presentation, mean (SD)^a^	72.8 (71.5)	98.8 (72.7)	.14
Albumin g/dL at presentation, mean (SD)^b^	3.4 (0.71)	3.2 (0.67)	.13
Mayo UC 3 endoscopic subscore, *n* (%)	60 (68.9)	18 (72)	.67

Abbreviations: anti-TNF, anti-tumor necrosis factor; BMI, body mass index; *C. difficile*, *Clostridioides difficile*; CMV, cytomegalovirus; IMM, immunomodulator; SD, standard deviation; UC, ulcerative colitis.

^a^Data missing on 16 patients.

^b^Data missing on 11 patients.

A similar proportion of patients in the early flexible sigmoidoscopy group were biologic-naïve compared to those in the delayed flexible sigmoidoscopy group (63% vs 64%; *P* = .94). *Clostridioides difficile* infection rates were similar in the early versus delayed flexible sigmoidoscopy groups (3.5% vs 4.0%, *P* = .89). The mean baseline CRP was 72.8 ± 71.5 mg/dL in the early flexible sigmoidoscopy group and 98.8 ± 72.7 mg/dL in the delayed flexible sigmoidoscopy group (*P* = .14). Mean baseline albumin levels were similar between the early and delayed flexible sigmoidoscopy groups (3.4 ± 0.71 g/dL vs 3.2 ± 0.67 g/dL, *P* = .13). The mean follow-up time was 27.9 ± 19.9 months in the early versus 26.9 ± 26.6 months in the delayed flexible sigmoidoscopy groups (*P* = .86).

### Outcomes: Unadjusted Analysis

Patients undergoing early flexible sigmoidoscopy had fewer mean days of IV corticosteroid use compared to those undergoing delayed flexible sigmoidoscopy (4.5 ± 3.6 vs 9.2 ± 10.5 days, *P* < .001). In addition, early flexible sigmoidoscopy was associated with decreased hospital length of stay (6.4 ± 4.2 vs 19.3 ± 17.6 days, *P* < .001).

**Figure 1.**
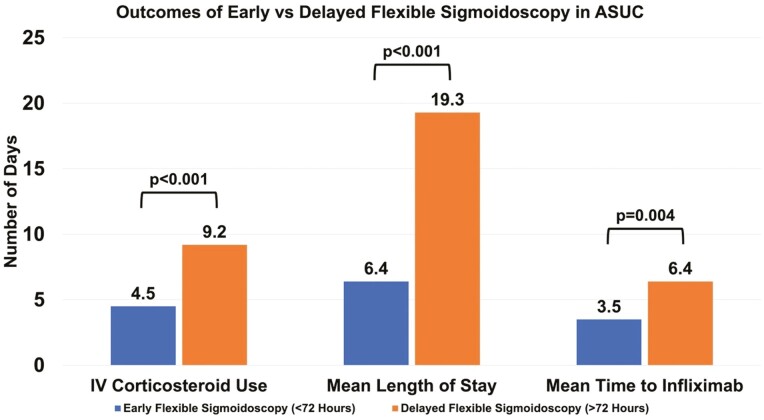
Outcomes of early versus delayed flexible sigmoidoscopy in acute severe ulcerative colitis.

**Figure 2.**
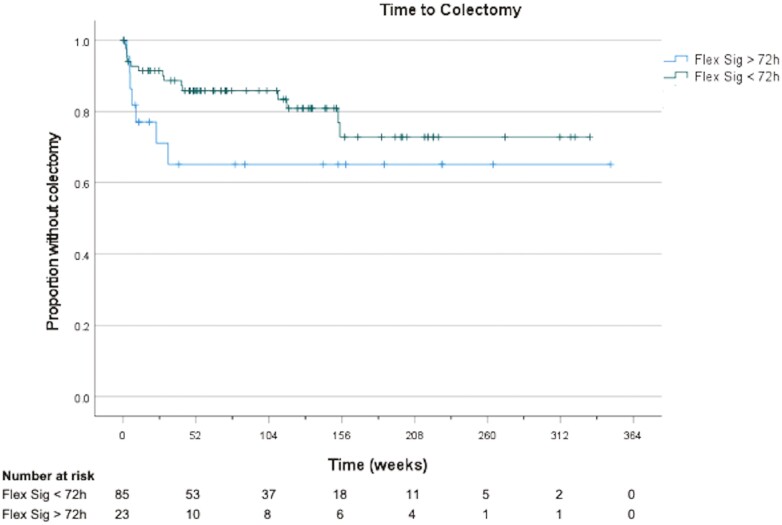
Kaplan–Meier curve showing time to colectomy in early versus delayed flexible sigmoidoscopy groups in acute severe ulcerative colitis.

In our cohort, infliximab was the only rescue medical therapy utilized. A total of 45% of patients (*n* = 40) in the early flexible sigmoidoscopy group and 48% of patients (*n* = 12) in the delayed flexible sigmoidoscopy group received rescue infliximab therapy (*P* = .86). Those who had an early flexible sigmoidoscopy had a shorter time to rescue infliximab therapy (3.5 ± 1.9 vs 6.4 ± 5.2 days, *P* = .004) (Figure 1). Compared to patients who underwent delayed flexible sigmoidoscopy, fewer patients who underwent early flexible sigmoidoscopy groups received inpatient opioid medications, though this did not reach statistical significance (50.6% vs 72.0%, *P* = .06). Similarly, there was a nonsignificant trend toward decreased colectomy rates in the early flexible sigmoidoscopy group (17% vs 28%, *P* = .23). Median time to colectomy was longer in the early compared to delayed flexible sigmoidoscopy group (1839 days vs 1618 days) but in unadjusted analysis, this did not reach statistical significance (log-rank *P* = .138) (Figure 2). A total of 8 patients in our cohort underwent colectomy during the index admission for ASUC (5 in the early flexible sigmoidoscopy group [5.7%] and 3 in the delayed flexible sigmoidoscopy group [12.0%]).

### Outcomes: Multivariate Regression Modeling

To further explore the impact on flexible sigmoidoscopy timing on relevant clinical endpoints including duration of IV steroids, we developed a multivariable model to adjust for baseline CRP, albumin, Mayo UC endoscopic subscore of 3, prior anti-TNF exposure, as well as use of oral corticosteroids on admission. Using a linear multivariate regression model, we found that each day that the flexible sigmoidoscopy was delayed was associated with approximately one additional day of IV corticosteroid use (adjusted coefficient = 1.008 [95% CI: 0.672–1.368], *P* < .001) (Table 2).

**Table 2.** Multivariate regression analysis of factors associated with duration of IV corticosteroid exposure^a^

Variables	Adjusted coefficient	95% CI	*P*-value
Prior anti-TNF exposure	−1.347	−4.128 to 1.435	.34
CRP at admission	0	−0.018 to 0.017	.96
Mayo UC subscore of 3 on endoscopy	2.164	−0.043 to 5.172	.16
Time to flexible sigmoidoscopy, days	1.008	0.672 to 1.368	**<.001**
Oral steroids on admission	2.319	−0.176 to 4.814	.07

Abbreviations: 95% CI, 95% confidence interval; anti-TNF, anti-tumor necrosis factor; CRP, C-reactive protein; IV, intravenous; UC, ulcerative colitis.

Bold values indicate *P*-value <.05.

^a^88 patients analyzed.

Multivariate analysis was performed to evaluate factors impacting time to infliximab salvage therapy. Increased time to flexible sigmoidoscopy was also independently associated with an increase in time to initiation of infliximab salvage therapy (adjusted coefficient = 0.664 [95% CI: 0.178–1.111], *P* = .008). In addition, we found that being on oral corticosteroids at the time of admission was associated with decreased time to infliximab salvage therapy (adjusted coefficient = −2.441 [95% CI: −4.530 to −0.353], *P* = .023) (Table 3).

**Table 3.** Multivariate regression analysis of factors associated with time to infliximab salvage therapy^a^

Variables	Adjusted coefficient	95% CI	*P*-value
Prior anti-TNF exposure	−1.985	−4.079 to 0.109	.06
CRP at admission	−0.004	−0.018 to 0.010	.53
Mayo UC subscore of 3 on endoscopy	−0.403	−2.788 to 1.983	.73
Time to flexible sigmoidoscopy, days	0.644	0.178 to 1.111	**.008**
Oral steroids on admission	−2.441	−4.530 to −0.353	**.023**

Abbreviations: 95% CI, 95% confidence interval; anti-TNF, anti-tumor necrosis factor; CRP, C-reactive protein; UC, ulcerative colitis.

Bold values indicate *P*-value <.05.

^a^40 patients analyzed.

Cox proportional hazards analysis was performed to evaluate the impact of time to flexible sigmoidoscopy on colectomy. After adjusting for baseline CRP, albumin, Mayo UC endoscopic subscore of 3, prior anti-TNF exposure, as well as immunomodulator and corticosteroid use on admission, we found that each day that the flexible sigmoidoscopy was delayed was independently associated with a 16% increase in risk of colectomy (hazard ratio [HR] = 1.160; 95% CI: 1.053–1.276; *P* = .002) (Table 4). We also found that oral corticosteroids at the time of admission was significantly associated with an increased risk of colectomy (HR = 3.693; 95% CI: 1.106–12.322; *P* = .034) (Table 4). As a sensitivity analysis, we limited our analysis to evaluate the impact of the time to flexible sigmoidoscopy on colectomy occurring during the index admission for ASUC and found similar results (HR = 1.238, *P* = .002).

**Table 4.** Cox proportional hazards analysis of risk of colectomy

Variables	Hazard ratio	95% CI	*P*-value
Prior anti-TNF exposure	0.838	0.244 to 2.880	.78
CRP at admission	1.001	0.993 to 1.009	.74
Mayo 3 findings on endoscopy	1.003	0.231 to 4.362	.74
Time to flexible sigmoidoscopy, days	1.160	1.053 to 1.276	**.002**
Oral steroids on admission	3.693	1.106 to 12.322	**.034**

Abbreviations: 95% CI, 95% confidence interval; anti-TNF, anti-tumor necrosis factor; CRP, C-reactive protein.

Bold values indicate *P*-value <.05.

### Supplementary Analyses

In supplementary analyses, using the cutoff point for an “early” flexible sigmoidoscopy as 24 hours, we found there was a numerically shorter duration of IV corticosteroid use, shorter length of hospital stay, and shorter time to infliximab although none of these were statistically significant (Supplementary Table 1).

In a similar model, flexible sigmoidoscopy performed within 48 hours of hospital admission was associated with a numerically shorter duration of IV corticosteroid use, and a statistically significant decrease in length of hospital stay, and shorter time to infliximab (Supplementary Table 2).

## Discussion

In this single-center retrospective study of 112 patients with ASUC who had a flexible sigmoidoscopy, we found that early flexible sigmoidoscopy (within 72 hours of admission) was independently associated with multiple favorable clinical outcomes including shorter duration of IV corticosteroid use, shorter length of hospital stay, and a shorter time to infliximab rescue therapy. There was also a decreased risk of colectomy on multivariate regression modeling in patients who underwent an early flexible sigmoidoscopy.

In ASUC, an expedient flexible sigmoidoscopy soon after hospital admission is essential. The roles of flexible sigmoidoscopy in this setting include evaluating endoscopic disease activity, which has prognostic value, and obtaining biopsies to rule out other etiologies such as CMV or ischemia. Multiple society guidelines and consensus statements have clearly recommended performing a flexible sigmoidoscopy in ASUC on admission, but few specified the optimal timing of the procedure.^12–14,17^ For example, the British Society of Gastroenterology guidelines recommends performing an “early” flexible sigmoidoscopy but does not specify a time point.^13^ On the other hand, the most recent ACG guidelines recommend performing a flexible sigmoidoscopy within 72 hours of admission (preferably within 24 hours).^14^ Our results support the recommendations of the ACG as we have demonstrated clinical benefit in patients with ASUC who had a flexible sigmoidoscopy within 72 hours of admission.

Two administrative database studies from the NIS have investigated the timing of endoscopy on outcomes in ASUC.^15,16^ Obi et al. reviewed NIS data from 2003 to 2016 and defined early endoscopy as being performed within 2 days of admission.^16^ The authors concluded that delayed endoscopy is associated with prolonged hospital stay, higher hospital cost, and mortality.^16^ Bali et al. conducted a similar study of the NIS from 2012 to 2018.^15^ In that study, early endoscopy was defined as flexible sigmoidoscopy or colonoscopy being performed within 48 hours of hospital admission.^15^ The authors noted that early endoscopy is associated with decreased length of hospital stay, healthcare utilization, and mortality. We demonstrated a similar finding of decreased hospital length of stay in our study. Patients with UC are at risk of hospitalization-related adverse events such as venous thromboembolism, and hospital-acquired infections due to immunosuppression.^18,19^ Therefore, a shorter hospital stay is an important outcome to reduce the burden of these adverse events.

Administrative database studies, however, have multiple limitations including the potential for miscoding of diagnoses which we avoid in our study by detailed individual chart review to confirm all diagnoses and procedures. The strengths of our study also include the availability of granular data such as previous and current medication use, lab results (CRP, albumin), and endoscopic findings that allow us to clearly define UC disease activity and severity. The two groups in our study had relatively similar markers of disease activity and severity including endoscopic disease activity as measured by the Mayo UC endoscopic subscore. We used the Truelove and Witts’ criteria to classify ASUC as it has been well-established to reflect disease severity. In addition, with access to granular data we were able to account and control for clinical confounders that can affect outcomes. Another strength of our study is the ability to account for other important clinical outcomes such as need for rescue infliximab, timing of infliximab, and need for colectomy.

In our study, we found a numerically lower rate of inpatient opioid use in the early flexible sigmoidoscopy group. Patients with IBD have a higher rate of opioid use disorder in general, and longer hospital courses with uncontrolled disease activity may be an important contributing factor to this problem.^20^ In patients with IBD, inpatient opioid administration has been linked to continued outpatient opioid medication use.^21^ Chronic outpatient opioid medication use in turn has been associated with a lower rate of compliance to biologic therapy and higher healthcare utilization.^22,23^ This highlights a potential negative impact of delayed flexible sigmoidoscopy in these patients.

Our results show that an earlier flexible sigmoidoscopy is associated with a shorter duration of IV corticosteroid use and a shorter time to infliximab therapy. Retrospective studies have shown that severe endoscopic lesions are associated with lack of response to corticosteroids and increased need for colectomy.^24,25^ An earlier assessment of endoscopic severity, and response (or lack thereof) to IV corticosteroids can expedite escalation to rescue therapy with biologics like infliximab. This could potentially improve long-term outcomes and colectomy rates.^26^ In our study, we were able to demonstrate a numerically lower rate of colectomy in the early flexible sigmoidoscopy group and a longer time to colectomy in those patients. These trends are similar to those seen from larger sample databases which also showed lower colectomy rates with early endoscopic evaluation.^15^

Concomitant *C. difficile* infection in UC has been shown to be associated with increased rates of poor clinical outcomes. A previously published systematic review revealed that the rate of *C. difficile* in inpatient UC ranges from 2.8% to 11.1%.^27–30^ The rate of *C. difficile* in our cohort was 3.6% (3.5% in the early vs 4% in the delayed flexible sigmoidoscopy groups), which is on the lower end of the published range. The differences in *C. difficile* rates can be partly attributed to testing assays/mechanisms. Our cohort utilized reflex *C. difficile* testing starting with glutamate dehydrogenase antigen and toxin A/B enzyme immunoassay followed polymerase chain reaction (PCR) toxin testing in discrepant results. This limits the false-positive rates of direct PCR testing and might account for our *C. difficile* rates being at the lower end of expected.

There are some limitations to our study. This includes the inherent limitations of retrospective studies such as missing data and lack of standardization. Another limitation is a relatively small sample size which may lead to inadequate power in determining statistical significance in certain outcomes. In addition, though the two groups were similar in terms of baseline disease activity and severity as measured by CRP, albumin, concomitant *C. difficile*/CMV infection, endoscopic Mayo UC score, and prior exposure to biological agents, residual confounders may have contributed to the delay in performing a flexible sigmoidoscopy.

In conclusion, in patients admitted with ASUC, early flexible sigmoidoscopy is associated with shorter duration of IV corticosteroid use, shorter duration of hospital stay, and shorter time to treatment escalation with infliximab. In addition, in multivariate analysis, we also found that increased time to flexible sigmoidoscopy was associated with an increased risk of colectomy. Our findings highlight the importance of performing a flexible sigmoidoscopy early (within 72 hours) during hospitalization to optimize outcomes for patients with ASUC.

## Supplementary Material

otad032_suppl_Supplementary_Tables

## Data Availability

Data are not publicly available. Deidentified data can be provided upon request to the corresponding author.
