# Supplementary material for: Early Flexible Sigmoidoscopy Improves Clinical Outcomes in Acute Severe Ulcerative Colitis
Source: Crohns Colitis 360. 2023 May 31;5(3):otad032. doi: 10.1093/crocol/otad032 (PMC10263117; doi:10.1093/crocol/otad032)
Supplement: otad032_suppl_Supplementary_Tables [file otad032_suppl_supplementary_tables.docx]

| Supplementary Table 1: Baseline Characteristics and Outcomes with 24 Hours Cut-off | | | |
| --- | --- | --- | --- |
| Variables | Flexible Sigmoidoscopy ≤ 24 hours (n=31) | Flexible Sigmoidoscopy > 24 hours (n=81) | p-value |
| *Patient characteristics* |  | | |
| Age (years), mean (SD) | 39.9 (17.3) | 39.0 (16.8) | 0.80 |
| Female, n (%) | 16 (51.6) | 35 (43.2) | 0.42 |
| BMI, mean (SD) | 26.9 (5.2) | 26.5 (7.2) | 0.78 |
| Current smoker, n (%) | 0 (0.0) | 6 (7.4) | 0.12 |
| On IMM on admission, n (%) | 1 (3.2) | 5 (6.2) | 0.54 |
| Biologic naïve, n (%) | 17 (54.8) | 54 (66.7) | 0.25 |
| Prior anti-TNF, n (%) | 12 (38.7) | 22 (27.2) | 0.23 |
| On chronic opioids, n (%) | 2 (6.5) | 6 (7.4) | 0.86 |
| On oral corticosteroids at time of admission, n (%) | 14 (45.2) | 42 (51.9) | 0.53 |
| *Disease activity* |  |  |  |
| Presence of extraintestinal manifestations (EIM), n (%) | 3 (9.7) | 6 (7.4) | 0.69 |
| Concomitant CMV infection, n (%) | 0 (0.0) | 3 (3.7) | 0.28 |
| Concomitant C. difficile infection, n (%) | 2 (6.5) | 2 (2.5) | 0.31 |
| C-reactive protein md/dL at presentation, mean (SD)* | 85.6 (74.7) | 77.2 (71.9) | 0.62 |
| Albumin g/dL at presentation, mean (SD)** | 3.5 (0.7) | 3.3 (0.7) | 0.18 |
| Mayo UC 3 Endoscopic Sub-Score, n (%) | 20 (64.5) | 58 (71.6) | 0.47 |
| *Outcomes* |  |  |  |
| Days of IV corticosteroid use, mean (SD) | 4.6 (4.1) | 5.9 (6.8) | 0.33 |
| Hospital length of stay, days, mean (SD) | 8.2 (11.8) | 9.7 (10.0) | 0.50 |
| Time to infliximab, days, mean (SD) | 3.6 (2.6) | 4.3 (3.4) | 0.34 |
| Colectomy, n (%) | 6 (19.4) | 16 (19.8) | 0.96 |
| *Data missing on 16 patients  **Data missing on 11 patients  *BMI: body mass index. IMM: immunomodulator; anti-TNF: anti-tumor necrosis factor; SD: standard deviation; CMV: cytomegalovirus; C. difficile: Clostridioides difficile; UC: ulcerative colitis* | | | |

| Supplementary Table 2: Baseline Characteristics and Outcomes with 48 Hours Cut-off | | | |
| --- | --- | --- | --- |
| Variables | Flexible Sigmoidoscopy ≤ 48 hours (n=63) | Flexible Sigmoidoscopy > 48 hours (n=49) | p-value |
| *Patient characteristics* |  | | |
| Age (years), mean (SD) | 38.8 (17.2) | 39.9 (16.6) | 0.73 |
| Female, n (%) | 33 (52.4) | 18 (36.7) | 0.10 |
| BMI, mean (SD) | 26.6 (5.6) | 26.7 (7.9) | 0.94 |
| Current smoker, n (%) | 3 (4.8) | 3 (6.1) | 0.75 |
| On IMM on admission, n (%) | 2 (3.2) | 4 (8.2) | 0.24 |
| Biologic naïve, n (%) | 38 (60.3) | 33 (67.3) | 0.44 |
| Prior anti-TNF, n (%) | 21 (33.3) | 13 (26.5) | 0.44 |
| On chronic opioids, n (%) | 4 (6.3) | 4 (8.2) | 0.71 |
| On oral corticosteroids at time of admission, n (%) | 32 (50.8) | 24 (49.0) | 0.85 |
| *Disease activity* |  |  |  |
| Presence of extraintestinal manifestations (EIM), n (%) | 5 (7.9) | 4 (8.2) | 0.97 |
| Concomitant CMV infection, n (%) | 1 (1.6) | 2 (4.1) | 0.42 |
| Concomitant C. difficile infection, n (%) | 3 (4.8) | 1 (2.0) | 0.44 |
| C-reactive protein md/dL at presentation, mean (SD)* | 75.9 (71.6) | 83.1 (73.8) | 0.63 |
| Albumin g/dL at presentation, mean (SD)** | 3.5 (0.7) | 3.2 (0.6) | 0.03 |
| Mayo UC 3 Endoscopic Sub-Score, n (%) | 43 (68.3) | 35 (71.4) | 0.72 |
| *Outcomes* |  |  |  |
| Days of IV corticosteroid use, mean (SD) | 4.7 (4.0) | 6.7 (8.1) | 0.09 |
| Hospital length of stay, days, mean (SD) | 6.4 (4.6) | 12.9 (14.2) | <0.001 |
| Time to infliximab, days, mean (SD) | 3.3 (2.1) | 5.1 (4.0) | 0.002 |
| Colectomy, n (%) | 14 (22.2) | 8 (16.3) | 0.44 |
| *Data missing on 16 patients  **Data missing on 11 patients  *BMI: body mass index. IMM: immunomodulator; anti-TNF: anti-tumor necrosis factor; SD: standard deviation; CMV: cytomegalovirus; C. difficile: Clostridioides difficile; UC: ulcerative colitis* | | | |
